# Functionalising Collagen-Based Scaffolds With Platelet-Rich Plasma for Enhanced Skin Wound Healing Potential

**DOI:** 10.3389/fbioe.2019.00371

**Published:** 2019-12-03

**Authors:** Ronaldo J. F. C. do Amaral, Noora M. A. Zayed, Elena I. Pascu, Brenton Cavanagh, Chris Hobbs, Francesco Santarella, Christopher R. Simpson, Ciara M. Murphy, Rukmani Sridharan, Arlyng González-Vázquez, Barry O'Sullivan, Fergal J. O'Brien, Cathal J. Kearney

**Affiliations:** ^1^Kearney Lab, Department of Anatomy and Regenerative Medicine, Royal College of Surgeons in Ireland (RCSI), Dublin, Ireland; ^2^Tissue Engineering Research Group (TERG), Department of Anatomy, Royal College of Surgeons in Ireland (RCSI), Dublin, Ireland; ^3^Centre for Research in Medical Devices (CURAM), National University of Ireland Galway, Galway, Ireland; ^4^Department of Biomedical Engineering, Khalifa University, Abu Dhabi, United Arab Emirates; ^5^Cellular and Molecular Imaging Core, Royal College of Surgeons in Ireland (RCSI), Dublin, Ireland; ^6^Advanced Materials and Bioengineering Research (AMBER) Centre, Dublin, Ireland; ^7^Centre for Research on Adaptive Nanostructures and Nanodevices (CRANN), Trinity College Dublin (TCD), Dublin, Ireland; ^8^Beaumont Hospital, Royal College of Surgeons in Ireland (RCSI), Dublin, Ireland; ^9^Trinity Centre for Bioengineering, Trinity College Dublin, Dublin, Ireland

**Keywords:** platelet-rich plasma, collagen-based biomaterial, skin wound healing, skin tissue engineering, scaffold vascularization, collagen-glycosaminoglycan scaffolds

## Abstract

Porous collagen-glycosaminoglycan (collagen-GAG) scaffolds have shown promising clinical results for wound healing; however, these scaffolds do not replace the dermal and epidermal layer simultaneously and rely on local endogenous signaling to direct healing. Functionalizing collagen-GAG scaffolds with signaling factors, and/or additional matrix molecules, could help overcome these challenges. An ideal candidate for this is platelet-rich plasma (PRP) as it is a natural reservoir of growth factors, can be activated to form a fibrin gel, and is available intraoperatively. We tested the factors released from PRP (PRPr) and found that at specific concentrations, PRPr enhanced cell proliferation and migration and induced angiogenesis to a greater extent than fetal bovine serum (FBS) controls. This motivated us to develop a strategy to successfully incorporate PRP homogeneously within the pores of the collagen-GAG scaffolds. The composite scaffold released key growth factors for wound healing (FGF, TGFβ) and vascularization (VEGF, PDGF) for up to 14 days. In addition, the composite scaffold had enhanced mechanical properties (when compared to PRP gel alone), while providing a continuous upper surface of extracellular matrix (ECM) for keratinocyte seeding. The levels of the factors released from the composite scaffold were sufficient to sustain proliferation of key cells involved in wound healing, including human endothelial cells, mesenchymal stromal cells, fibroblasts, and keratinocytes; even in the absence of FBS supplementation. In functional *in vitro* and *in vivo* vascularization assays, our composite scaffold demonstrated increased angiogenic and vascularization potential, which is known to lead to enhanced wound healing. Upon pro-inflammatory induction, macrophages released lower levels of the pro-inflammatory marker MIP-1α when treated with PRPr; and released higher levels of the anti-inflammatory marker IL1-ra upon both pro- and anti-inflammatory induction when treated with the composite scaffold. Finally, our composite scaffold supported a co-culture system of human fibroblasts and keratinocytes that resulted in an epidermal-like layer, with keratinocytes constrained to the surface of the scaffold; by contrast, keratinocytes were observed infiltrating the PRP-free scaffold. This novel composite scaffold has the potential for rapid translation to the clinic by isolating PRP from a patient intraoperatively and combining it with regulatory approved scaffolds to enhance wound repair.

## Introduction

Porous collagen-glycosaminoglycan (collagen-GAG) scaffolds were amongst the first scaffolds developed for tissue engineering applications (Yannas et al., 1982). These scaffolds are produced by combining collagen type I with glycosaminoglycan followed by a controlled freeze drying procedure, resulting in highly porous and biomimetic scaffolds (O'brien, 2011). These scaffolds readily allow for cell infiltration and their degradation is balanced by the formation of new tissue by the infiltrating cells. Their use has seen widespread positive clinical results in treating burns (Heimbach et al., 1988), scar contractures (Stiefel et al., 2010), and diabetic foot ulcers (DFU) (Driver et al., 2015), and it is currently FDA-approved for these applications. Nevertheless, their use in skin wound healing involves a two-stage surgical procedure and a long period for total wound healing. Collagen-GAG scaffolds are typically implanted at the wound site with an outer silicone layer. After 21 days, which is the period necessary for the dermal layer to vascularise, the outer silicone layer is removed and replaced by an ultrathin epidermal autograft, which will lead to full lesion healing in approximately 30 days (Shahrokhi et al., 2014). Reducing the duration of healing and/or eliminating the requirement for a second procedure would be a clear advantage clinically, reducing infection risk, costs, and enhancing patient welfare.

To reduce the healing time, strategies that could accelerate the vascularization of the dermal layer would provide great benefit. Indeed, the incorporation of recombinant growth factors, genes or other extracellular matrix (ECM) components into these scaffolds could improve their healing potential (Matsiko et al., 2015; Quinlan et al., 2017; Laiva et al., 2018). Additionally, we hypothesized that filling the pores of the scaffold with ECM would present a continuous upper surface for keratinocytes to repopulate. We identified platelet-rich plasma (PRP) as a cost-effective, autologous, intraoperatively available source of a variety of growth factors that could be incorporated into the pores of the collagen-GAG scaffold. Furthermore, the PRP can be activated with calcium to form a clot within the pores of the scaffold, providing a surface for re-epithelialization.

PRP is a blood-derived product, processed to yield platelets in high concentration in plasma. Upon activation (e.g., with CaCl_2_), it releases growth factors in high concentrations (Foster et al., 2009; Amable et al., 2013). Many of the factors released play important roles in wound healing and angiogenesis/vascularization, including: transforming growth factor beta (TGF-β), platelet-derived growth factor (PDGF), vascular endothelial growth factor (VEGF) and fibroblast growth factor (FGF) (Werner and Grose, 2003; Barrientos et al., 2008). Indeed, a serum containing growth factors released from PRP (PRP releasate or PRPr) can modulate cell phenotype and potentially substitute the need for fetal bovine serum (FBS) in human cell culture medium (Amable et al., 2014; Rubio-Azpeitia and Andia, 2014; Do Amaral et al., 2015, 2016). When PRP is activated a gel or clot is formed and PRP gel has shown positive clinical outcomes for wound healing as a point-of-care autologous therapeutic, i.e., patient blood is collected, processed for PRP gel acquisition and implanted in a single-stage at bedside (Frykberg et al., 2010; Lacci and Dardik, 2010; Carter et al., 2011). However, many reports present inconsistent and inconclusive results (Martinez-Zapata et al., 2016). Suggested drawbacks of using PRP directly as a gel include the mismatch between native skin and PRP gel's mechanical properties (Sell et al., 2012) and the undesirable burst release profile of the growth factors, which would likely be more effective if delivered in a sustained manner from a biomaterial (Xie et al., 2014). In this regard, collagen scaffolds have been shown to improve the mechanical properties of (non-PRP derived) fibrin gels (Brougham et al., 2015), and, under certain processing conditions, to control the release of growth factors (Matsiko et al., 2015).

Consequently, we hypothesized that the incorporation of PRP into the collagen-GAG scaffolds would provide the scaffolds with growth factors to enhance their regenerative capacity, while the scaffolds would provide PRP with mechanical stability and promote a more controlled release of the growth factors. The mechanically stable PRP clot could additionally provide a surface for keratinocyte seeding. The overall objective of this work was to develop fabrication techniques for a novel composite scaffold that combines an off-the-shelf scaffold (collagen-GAG) with a natural source of growth factors—PRP—and to keep these techniques simple enough to be replicated in surgery prior to implantation. In addition, we tested the composite scaffold's potential to enhance wound healing by accelerating vascularization and wound closure. Specifically, after incorporating PRP into collagen-GAG scaffolds, we analyzed: the structural characteristics of the novel composite; the release of growth factors; the effects on angiogenesis and vascularization *in vitro* and *in vivo*; the effects on macrophage polarization; and the capacity to support a co-culture of keratinocytes and fibroblasts *in vitro*.

## Materials and Methods

### Platelet-Rich Plasma Acquisition

PRP was obtained from human buffy coat samples (*n* = 40 different healthy donors) obtained from the Irish Blood Transfusion Centre in accordance with the Royal College of Surgeons in Ireland Research Ethics Committee (REC1463b). Briefly, buffy coat was separated into 50 mL tubes and centrifuged at 600 × g for 5 min at room temperature. The upper phase was collected and platelets were counted using a hematology analyser (Sysmex, Kobe, Japan). Platelet count was then adjusted to a desired concentration of 10^6^/μL, using plasma. To form the PRP gel, CaCl_2_ solution was added to PRP samples up to a final concentration of 20 mM. Samples were incubated at 37°C for 1 h for gel formation. For some *in vitro* studies, PRP gel was further incubated overnight at 4°C. Finally, samples were centrifuged at 2,000 × g for 5 min. The supernatant/serum or PRP in liquid form, also known as PRP releasate (PRPr) was collected and stored at −20°C prior to use (Do Amaral et al., 2015).

### Scaffold Fabrication and PRP Incorporation

In order to fabricate collagen-GAG scaffolds, microfibrillar type I bovine tendon collagen (Integra Life Sciences, Plainsboro, NJ) was blended with chondroitin-6-sulfate, isolated from shark cartilage (Sigma–Aldrich, Germany) in 0.05 M acetic acid and freeze-dried as previously described (O'brien et al., 2005; Murphy et al., 2010; Do Amaral et al., 2019). Briefly, the suspension was frozen to a final temperature of −10°C, which was maintained constant for 60 min, and then sublimated under vacuum (100 mTorr) at 0°C for 17 h. The resulting porous scaffolds were cut into discs of 4 × 6 mm, physically cross-linked by a dehydrothermal treatment (DHT) using a vacuum oven (Vacucell, MMM Group, Munich, Germany) at 0.05 bar and 105°C over 24 h, followed by a chemical crosslinking using 1-ethyl-2-(3-dimethylaminopropyl) carbodiimide (EDAC) in combination with N-hydroxysuccinimide (NHS) as previously described. Scaffolds were then sterilized in 70% ethanol and stored in sterile phosphate buffered saline (PBS). In order to incorporate PRP, collagen-GAG scaffolds were previously incubated in 50 mM CaCl_2_ solution for 20 min at room temperature, and then incubated in PRP for 1 h at 37°C, after which a composite scaffold was obtained. Changes in PRP gel, collagen-GAG and composite scaffolds' disc area and circularity as a function of time (time zero, 1 day and 3 days) were measured with ImageJ software and statistical significance was evaluated by Two-way Anova with Bonferroni's post-tests. Of note, our time zero in this case correspond to scaffolds at the end of production (i.e., after the 1 h period necessary for fibrin polymerization).

### Mechanical Testing

To analyse whether the composite scaffold had enhanced mechanical properties over PRP clots, collagen-GAG scaffolds, PRP gel and our composite scaffold had their mechanical properties evaluated by uniaxial tensile tests (*n* = 5). All samples were produced in a dog-bone shape of 63.5 mm long with a narrowed center of 3 mm as per ASTM D638 (specimen type V) and were tested using a mechanical testing machine (Zwick/Roell, Ulm, Germany) fitted with a 5N load cell, being kept hydrated in PBS throughout the testing. The tests were conducted at a strain rate of 20% per minute. The modulus was defined as the slope of a linear fit to the stress–strain curve over 20–25% strain. Elongation was characterized as the strain (%) at maximum stress. Statistical significance was determined by One-way Anova with Tukey's Multiple Comparison Test.

Tribology studies were also performed in both collagen-GAG scaffolds and our composite scaffold using a Discovery HR-2 Rheometer (TA instruments) (*n* = 3) to evaluate the materials adhesiveness, which is an important feature for skin tissue engineered constructs (Salamone et al., 2016; Qu et al., 2018). Eight millimeter diameter steel parallel plate and grip topography were used. Temperature was kept constant 37°C with no soaking time. Run was performed on a sweep increased speed from 0.05 rad/s in 16 steps up to 50 rad/s. The gap between the attachment and heating plate was kept constant and torque was collected and analyzed on the angular velocity. Linear regression analysis was performed to asses if the composite scaffold had more friction than the collagen-GAG scaffolds in a dry or PBS-wet state. Of note, our protocol to produce the composite scaffolds results in scaffolds that are inherently wet. On the other hand, the collagen-GAG scaffolds are obtained dry after freeze-drying process. Therefore, in order to better compare with the wet composite scaffolds, we also tested PBS-wetted collagen-GAG scaffolds.

### Histological Analysis

To evaluate PRP-derived fibrin distribution within the novel composite scaffolds, histological staining, immunofluorescence and scanning electron microscopy (SEM) analysis were performed. Collagen-GAG scaffolds, PRP gel and our composite scaffolds were fixed in 10% neutral buffered formalin solution (Sigma-Aldrich, Ireland) for 24 h at 4°C. For histological staining (*n* = 4), fixed samples were embedded in paraffin and 5 μm thick slices were obtained after microtoming with a Leica RM2255 microtome. After hydration steps, samples were stained with Masson's Trichrome (HT15-1KT Sigma-Aldrich) according to manufacturer with the exclusion of hematoxylin as no cells were present in these samples. In the Masson's trichrome staining, aniline blue could detect mainly the collagen-GAG, while biebrich scarlet-acid fuchsin could detect PRP-derived fibrin. Images were taken after observation with a light microscope (ECLIPSE 90i; Nikon, Tokyo, Japan) equipped with DS-Ri1 camera and NIS elements software (Nikon). Immunofluorescence followed by confocal analysis (*n* = 3) was performed to specifically observe fibrinogen distribution. Fixed samples were washed in PBS and unspecific antigen block and cell permeabilization was performed with 0.3% triton 100x (Sigma-Aldrich, Ireland) + 3% fetal bovine serum (FBS) in PBS for 20 min. The presence of fibrin was detected using mouse monoclonal anti-fibrinogen antibody (ThermoFisher Scientific MA5-15906) diluted at 1:200 in PBS + 1% BSA incubated overnight at 4°C, followed by incubation with a rat anti-mouse IgG (H + L) secondary antibody, FITC (eBiosciences, 11401185) diluted at 1:100 in PBS + 1% BSA for 1 h at room temperature. Importantly, the anti-fibrinogen antibody that we used targets specifically the gamma component of fibrinogen, which is still present after polymerization into fibrin (Mosesson, 2005). Therefore, we did not correlate this staining with levels of PRP activation. For cytoskeleton staining, samples were incubated in phalloidin TRTC (Sigma) at 1:600 diluted in PBS for 20 min. For nuclei staining, Hoechst 33258 at 1:10,000 diluted in PBS was added for 5 min. Samples were observed transversely using a Carl Zeiss LSM 710 confocal microscope equipped with either a W Plan-Apochromat 20x (N.A. 1.0) or W N-Achroplan 10x (N.A. 0.3) objective. Z stack images were acquired 30 μm below the surface to yield a 150 μm total depth. One central and two peripheral images were acquired per sample. Finally, to observe fibrin distribution and the collagen-GAG strut at the ultrastructure level, samples were observed under the SEM. Samples (*n* = 3) were prepared by plunging into liquid nitrogen for 10 s and sublimed for 1 h under vacuum. The samples were then sectioned and sputter coated before imaging at 2 kV in the SEM (Carl Zeiss Ultra, Germany).

### Growth Factor Release

To evaluate growth factor release, PDGF-BB, bFGF, VEGF and TGF-β1 released from PRP gel and our composite scaffold were quantified by ELISA assays (DY220, DY233, DY293B, DY240, all from R&D systems) (*n* = 6). We chose PRP gel as control samples to our composite scaffold for this experiment since collagen-GAG scaffolds do not possess growth factors. Total protein release was quantified with Quick Start™ Bradford Reagent (Bio-Rad). We also did not use collagen-GAG as control samples for total protein release, since the physical and the chemical cross-linking that we performed make the collagen-GAG scaffolds highly resistant to degradation. It has been shown that with these cross-linking steps, these scaffolds remain practically intact even after 1 week exposure to collagenase and chondroitinase (Pek et al., 2004). Therefore, the contribution of collagen-GAG scaffold-derived proteins to total protein quantification in this assay can be considered irrelevant. Statistical significance was evaluated by Two-way Anova with Bonferroni's post-tests. To compare groups, the growth factors and total protein release levels were normalized to the volume of PRP used to prepare the sampled. After 1 h incubation at 37°C to activate platelets, composite scaffolds and PRP gel samples were transferred to 48-well-plates and incubated with 1 mL Endothelial Basal Medium-2 (EBM-2, Lonza) per sample. At this point and after 1 h, 1, 4, 7, and 14 days, 500 μL medium sample was collected, frozen, and 500 μL fresh medium was added to the wells. Samples were kept at humidified atmosphere of 5% CO_2_ at 37°C throughout the 14 days of experiment.

### Cell Culture

Human mesenchymal stromal cells (hMSC) (*n* = 3 different donors) were isolated from bone marrow aspirates obtained from the iliac crest of normal human donors 20–30 years old (Lonza Biologics PLC) as previously described (Barreto et al., 2017). Pooled Human Umbilical Vein Endothelial Cells (HUVEC) (CC-2519; Lonza Switzerland), human keratinocyte cell line (HACAT) (300493; CLS Cell Lines Service GmbH, Germany), and monocyte derived macrophages (THP1) (TIB-202, ATCC) were commercially purchased. Human dermal fibroblasts (BJ) were kindly donated by The Garlick Lab, USA. Cells were cultured in standard tissue culture flasks and incubated at 37°C under humidified atmosphere of 5% CO_2_ and 95% relative humidity. hMSC and BJ were cultured in low glucose Dulbecco's modified Eagle's medium (DMEM) supplemented with 10% FBS (Hyclone); HUVEC in Endothelial Growth Medium-2 (EGM-2, Lonza, Switzerland); and HACAT in Optimized DMEM (Caltag Medsystem, UK) supplemented with 10% FBS (Hyclone). THP1 were cultured in RPMI-1640 R8758 supplemented with 10% FBS (Hyclone), 10 mM 4-(2-hydroxyethyl)-1-piperazineethanesulfonic acid (HEPES), 1 mM sodium pyruvate, 2.5 g/L glucose, and 0.05 mM mercaptoethanol (Sigma). Medium was replaced twice a week and upon reaching 80–90% confluency, cells were passaged using trypsin–EDTA solution. hMSC, HUVEC and THP1 were used up to passage 6 in all experiments. HACAT and BJ were used up to passage 30.

### Cell Proliferation and Migration

To analyse proliferation, cell DNA content was quantified with Quant-iT PicoGreen dsDNA assay (Thermo Fisher Scientific, Biosciences, Ireland) according to manufacturer's instructions after 96 h of culture. For evaluation of cell migration, a scratch assay was performed in confluent 24 well-plates using P20 micropipette tips. Phase contrast images were acquired after 16 h with Leica DMIL microscope equipped with Leica DFC420 C camera and wound closure was quantified using ImageJ software. For proliferation and migration assays, medium without FBS but supplemented with PRPr (*n* = 3) instead were also tested. Finally, to analyse the effect of our composite scaffolds on cell proliferation and migration capacity, our composite scaffolds (*n* = 3) were placed in Millicell Hanging Cell Culture Insert of 8 μm porosity (Merck Millipore, Ireland), while cells were cultured in medium without FBS in the wells below. Statistical significance was evaluated by One-way Anova with Dunnett's Multiple Comparison Test comparing all samples with their respective FBS-supplemented control.

### Tubule Forming Assay

To analyse angiogenesis *in vitro*, a tubule formation assay was performed with HUVECs seeded in Matrigel® (Corning). Briefly, our composite scaffolds (*n* = 4) were incubated with Endothelial Basal Medium (EBM) or EGM-2 without FBS supplementation (1 mL/scaffold) for 24 h at 37°C. 120 μL/well of Matrigel® was added to 48 well-plates and allowed to solidify at 37°C for 30 min. 3 × 10^4^ HUVEC were seeded per well and the 500 μL conditioned medium from the composite scaffolds was added. Therefore, each scaffold conditioned medium was used in two wells of the 48 well-plates. Moreover, EBM and EGM-2 without FBS but supplemented with PRPr (*n* = 3) instead were also tested. EBM and EGM-2 supplemented with 2% FBS were used as control. Images were taken after 3, 7, 24, 48, and 72 h and intersections points; number of tubes; total tube length; and average tube length were measured using Image J software. Statistical significance was evaluated by One-way Anova with Dunnett's Multiple Comparison Test comparing all samples with their respective FBS-supplemented control at each time-point.

### Vascularization Assay

To evaluate the scaffolds capacity to be vascularised *in vitro*, a co-culture system of HUVEC and hMSC in a ratio of 4:1, respectively, with 5 × 10^5^ cells in total was performed as previously described, with minor modifications (Duffy et al., 2011; Mcfadden et al., 2013; Lloyd-Griffith et al., 2015; Do Amaral et al., 2019). HUVEC were initially seeded with hMSC added to the culture 3 days after. Scaffolds were cultured in 24-well-suspension plates in 2 ml medium. Some collagen-GAG scaffolds were cultured in EGM-2 medium supplemented with 2% FBS up to the first 3 days of culture, later changed to 20% FBS when hMSC were added. Other scaffolds were cultured in EBM-2 medium without FBS but supplemented with 2% PRPr (*n* = 6) instead, later changed to 5% PRPr after 3 days. Our composite scaffolds (*n* = 6) were always kept in EBM-2 without FBS. For HUVEC seeding in the composite scaffolds, HUVECs were mixed in PRP prior to PRP incorporation into the collagen-GAG scaffolds. After 10 days of initial HUVEC seeding samples were fixed in 10% neutral buffered formalin solution (Sigma-Aldrich, Ireland), following by immunofluorescence staining with phalloidin-TRTC (Sigma) at 1:600 and nuclei counter staining with Hoechst 33258 at 1:10,000. Samples were then sliced in half and observed transversely using a Carl Zeiss LSM 710 confocal microscope equipped with a W N-Achroplan 10× (numerical aperture 0.3) objective. Three images were taken per sample, in which 1 was from the center and 2 from the periphery. Vessel-like structures were manually counted and automatically measured (area and ferret) using an in-house developed and publicly available ImageJ macro (Do Amaral et al., 2019). Statistical significance was determined by One-way Anova with Tukey's Multiple Comparison Test.

### Chick Chorioallantoic Membrane (CAM) Assay

CAM assay was used to investigate scaffolds' angiogenic potential *in vivo*, as previously described (Ryan et al., 2019). All experimentation carried out on chick embryos was in accordance with the EU Directive 2010/63/EU for animal experiments. Briefly, fertilized chicken eggs (*n* = 6) at day 0 of development (Ovagen Group Ltd, Co. Mayo, Ireland) were incubated at 37°C for 3 days, when they were cracked and placed into 100 mm diameter petri dishes (Corning Inc., New York, USA). These were placed inside a larger petri dish of 150 mm diameter (Corning Inc., New York, USA) containing PBS, as a humidified chamber. The chicks were further incubated for 4 days, completing 7 days of development, when collagen-GAG scaffolds soak loaded in PBS and our composite scaffolds were placed on the CAM membrane. We chose collagen-GAG scaffolds as controls for our composite scaffolds in this experiment in order to test the hypothesis that PRP incorporation would enhance the angiogenic potential of collagen-GAG scaffolds. After an additional 5 days of incubation (development day 12) the samples were imaged. The vascularization around the scaffolds was quantified using ImageJ software after image treatment with the “Mexican Hat Filter,” conversion to 8-bit and measurement of blood vessel area. Statistical significance was determined by unpaired two-tailed *t*-test.

### Macrophage Polarization Assay

To analyse the effects of our scaffolds on macrophages, THP1 were seeded in 24 well-plates at a density of 125 × 10^3^/cm^2^. Twenty nanogram per milliliter PMA was added to culture media to promote monocyte to macrophage transition and cells were incubated overnight. After 16 h, fresh growth medium was added after removal of the PMA, and after 6 h cells were induced to M0, M1, or M2 phenotype. M0 comprised of regular growth medium, M1 media was supplemented with 5 ng/mL interferon-γ (IFNγ) and 100 ng/mL lipopolysaccharide (LPS), and M2 media was supplemented with 20 ng/mL of interleukin-4 and -13 (IL4 and IL13) (all from Sigma). Experimental groups comprised of cell culture media supplemented with 1% PRPr, 2.5% PRPr, 5% PRPr, 10% PRPr or our composite scaffolds placed in Millicell Hanging Cell Culture Insert of 8 μm porosity (Merck Millipore) in substitution to 10% FBS (*n* = 3 each). After 72 h in culture the cell culture media was collected for analysis of Macrophage Inflammatory Protein (MIP-1α), Tumor Necrosis Factor-α (TNFα), and Interleukin-1 Receptor Antagonist (IL1-ra) secretion by ELISA (eBioscience, Ireland) (Sridharan et al., 2018). Statistical significance was evaluated by One-way Anova with Dunnett's Multiple Comparison Test comparing all samples with their respective FBS-supplemented control in each condition (M0, M1, and M2).

### 3D Fibroblast and Keratinocyte Co-culture

A co-culture of fibroblasts and keratinocytes was performed *in vitro*, to examine the scaffolds' capacity to support these cells and therefore promote wound healing. 5 × 10^5^ BJ were initially seeded in 6 mm diameter collagen-GAG scaffolds (*n* = 3). For our composite scaffolds (*n* = 3), BJ were mixed in PRP prior to incorporation into the collagen-GAG scaffolds. After 2 days in culture, 1.4 × 10^6^ HACAT were seeded on top of the scaffolds, which were transferred to the air liquid interface by reducing the volume of medium to 300 μL in a 24-well-plate. At this point, optimized DMEM was used instead of DMEM low glucose. One hundred microliter of media was changed per sample every day. Of note, cell culture medium in our composite scaffolds was always kept without FBS, while cell culture medium of collagen-GAG scaffolds was supplemented with 10% FBS. After 14 days at the air liquid interface, samples were fixed in 10% formalin and embedded in paraffin for histological analysis. After 5 μm microtoming slicing and placement on glass slides, samples were stained with hematoxylin & eosin (H&E) (Sigma). In order to further identify the keratinocytes and the fibroblasts in the scaffolds, paraffin-embedded samples were deparaffinised, antigen was retrieved with citrate buffer steam and cytokeratin was identified with monoclonal anti-pan cytokeratin antibody clone PCK-26 (Sigma) (dilution 1:100) and goat anti-mouse IgG H&L Alexa Fluor 568 antibody (Abcam) (dilution 1:500). Cell nuclei were stained with DAPI (Sigma) (dilution 1:2,000). Images were taken after observation in fluorescence microscope (ECLIPSE 90i; Nikon, Tokyo, Japan) equipped with DS-Ri1 camera and NIS elements software (Nikon).

### Statistical Analysis

Statistical analysis was performed using GraphPad Prism 5.0 software and statistical significance was considered when *p* < 0.05. Different statistic tests were used for specific experiments as described in their respective methodology sections. In some figures we used ^*^ to denote *p*-values, in which ^*^p < 0.05, ^**^p < 0.01, and ^***^p < 0.001.

## Results

### PRP Can Be Homogenously Incorporated Into Collagen-GAG Scaffolds

**Figure 1:**
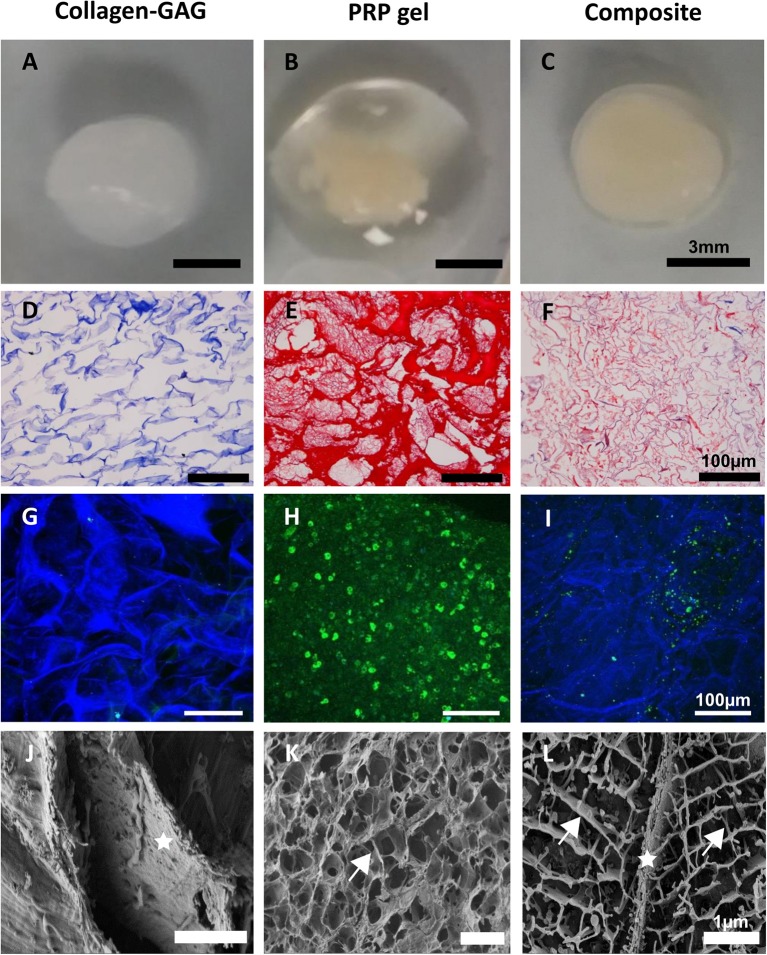
Platelet-rich plasma (PRP) was successfully incorporated into collagen-GAG scaffolds. Collagen-GAG scaffolds **(A,D,G,J)**, PRP gel **(B,E,H,K)** and the composite scaffold **(C,F,I,L)** were visualized by naked eye **(A–C)**, histology **(D–F)**, immunofluorescence **(G–I)**, and scanning electron microscopy **(J–L)**. PRP-derived fibrin was evenly incorporated within the pores of collagen-GAG scaffolds. Aniline blue (blue) evidencing collagen **(D,F)** and Biebrich Scarlet-Acid Fucshin (red) evidencing fibrin **(E,F)**. Collagen-GAG strut autofluoresced in blue **(G,I)** and fibrinogen immunostating in green **(H,I)**. Star indicates the collagen-GAG strut in **(J,L)**, and arrows indicates the fibrin network in **(K,L)**.

In testing our objective of incorporating PRP within the collagen-GAG scaffolds, the structural analysis of the original collagen-GAG scaffold, PRP gel, and the novel composite scaffolds suggests such incorporation was efficiently achieved (Figure 1). While PRP gel alone did not present a defined shape (Figure 1B), our composite scaffolds (Figure 1C) acquired the same shape as the collagen-GAG scaffolds (Figure 1A). Moreover, it is possible to observe a large volume of liquid released from the PRP gel, which is indicative of the lack of a controlled release of PRP content in the gel after activation (Figure 1B). On histological analysis, the collagen-GAG scaffolds presented a porous architecture as expected (Figure 1D). For the PRP gel, there is a heterogeneous distribution of fibrin in the gel with some thin fibers and other thick deposits (Figure 1E). In our composite scaffolds, the PRP-derived fibrin was found filling the pores throughout the collagen-GAG structure (Figure 1F), with thin fibers more homogenously distributed within the pores of the composite scaffold than in the PRP gel. Fibrin incorporation within the pores of the collagen-GAG scaffolds could also be observed through immunofluorescence (Figures 1G–I), confirming the histological observations (SEM) (Figures 1J–L). Finally, SEM analysis shows the collagen-GAG strut (Figure 1J) and the fibrin network in PRP gel (Figure 1K), which is homogenously incorporated within the pores of a well-preserved collagen-GAG strut in our composite scaffolds (Figure 1L).

### Composite Scaffolds Match the Mechanical Properties of Collagen-GAG Scaffolds and Exceeds That of the PRP Gel

**Figure 2:**
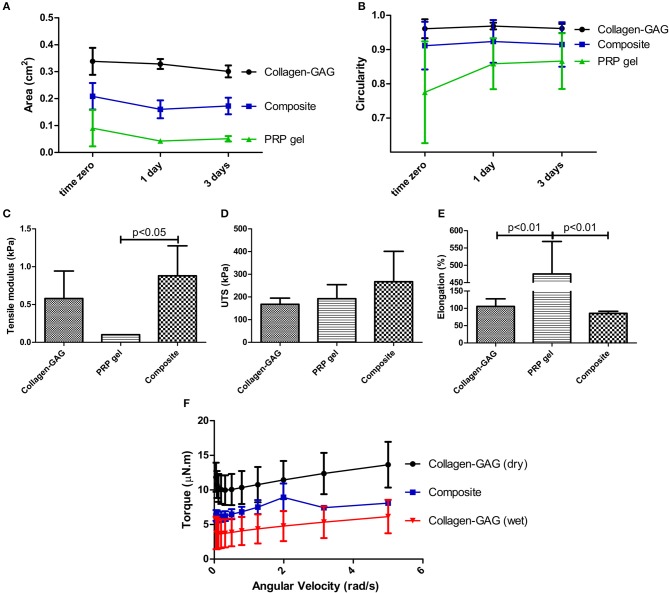
Composite scaffolds acquired the mechanical properties of collagen-GAG scaffolds. Area **(A)** and circularity **(B)** of the samples showed that the composite scaffolds acquired a similar shape compared to collagen-GAG scaffolds, as well as mechanical properties, as observed in tensile modulus **(C)**, ultimate tensile strength **(D)**, and elongation **(E)**. Rheometry shows that the composite scaffold had increased torque values compared to wet collagen-GAG scaffolds at the range of angular velocity tested **(F)**. Data are expressed as mean ± standard deviation. Samples had significant different areas **(A)** at all time-points; and significant differences in circularity **(B)** were observed between PRP gel samples compared to both collagen-GAG and composite scaffolds at time zero, as determined after two-way Anova with Bonferroni post-test analysis. Tensile modulus **(C)**, ultimate tensile strength **(D)**, and elongation statistical analysis was performed with one-way Anova with Tukey's Multiple Comparison Test.

One of the main drawbacks of the use of PRP gel on its own are its poor mechanical properties, and we hypothesized that incorporation into collagen-GAG scaffolds could resolve that issue. Indeed, PRP gel contracts in just 1 h after activation. Such contraction is partially avoided when PRP is incorporated into the collagen-GAG scaffolds forming our composite scaffolds, as observed by measuring scaffold area (Figure 2A). At the time-points analyzed, collagen-GAG scaffolds area was 1.6–2 times bigger than our composite scaffolds, while the composite area was 2.3–4 times bigger than PRP gel area, which demonstrates the reduction in contraction. Such differences in the area of the analyzed samples were statistically significant at all time-points. On the other hand, circularity was measured to quantify the materials shape, specifically, how close they are to a perfect circle (i.e., the starting shape; circularity value of 1). It was then possible to verify that the composite scaffolds acquired a statistically similar circular shape of the collagen-GAG scaffolds already at 1 h post-PRP activation (time zero) (Figure 2B). By contrast, PRP gel was statistically less circular compared to the composite scaffold and collagen-GAG scaffolds at time zero. Only after 1 and 3 days did the PRP gel acquire circularity statistically similar to the other materials, probably because contraction overtime leads to the formation of a more circular, albeit smaller, gel.

Most importantly, the PRP gel's resistance to tension is very low, as observed in tensile modulus analysis (Figure 2C), even though all tested materials presented similar ultimate tensile strength (Figure 2D). Indeed, the composite scaffold's tensile modulus matched the collagen-GAG scaffold (Figure 2C). The PRP gel did have a large elongation capacity, which was not observed in the collagen-GAG or composite scaffolds (Figure 2E). These data demonstrate that incorporation of PRP into the collagen-GAG scaffolds results in a composite scaffold with similar mechanical properties compared to the original collagen-GAG scaffolds.

Finally, tribological analysis (Figure 2F) revealed the composite scaffold's ability to generate increased friction, suggesting an increased adhesiveness, compared to wet collagen-GAG scaffolds in PBS, but still below the high friction generated from dry collagen-GAG scaffolds (intercepts *p* < 0.0001). Indeed, since skin is submitted to tension from different directions, adhesiveness is considered an important feature for skin tissue engineered constructs (Salamone et al., 2016; Qu et al., 2018). Importantly, the slopes of the linear regression analysis were not different, suggesting that PRP incorporation did not change the overall characteristics' of the collagen-GAG scaffolds, whilst conferring a property of increased observed friction. Due to its amorphous shape and its contractile characteristic, it was not possible to measure the PRP gel in the tribology analysis.

### PRP-Derived Growth Factors Were Released From Collagen-GAG Scaffolds for Up to 14 Days

**Figure 3:**
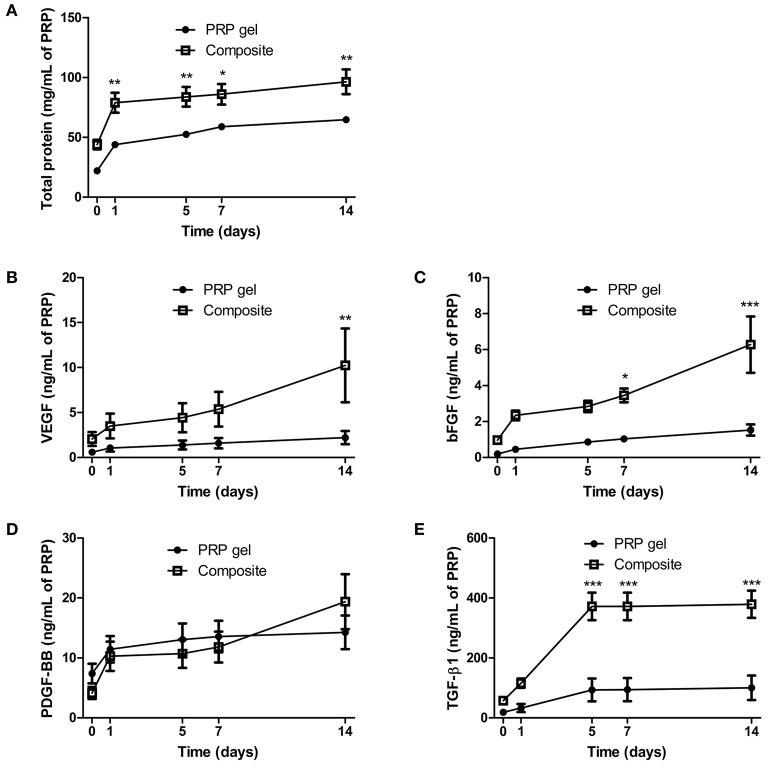
Cumulative release of proteins and growth factors was observed for up to 14 days in composite scaffolds. PRP-derived proteins **(A)** and growth factors, particularly VEGF **(B)**, bFGF **(C)**, PDGF-BB **(D)**, and TGF-β1 **(E)**, were released from PRP gel and the composite scaffold for 14 days. Data are expressed as mean ± standard error of the mean. *p < 0.05, **p < 0.01, and ***p < 0.001 after two-way Anova with Bonferroni post-test analysis.

After successful incorporation of PRP into collagen-GAG scaffolds, we next investigated whether the incorporated PRP would provide the scaffold with growth factors that could enhance their regenerative properties, and whether the collagen-GAG scaffolds would affect the release of PRP-derived growth factors. Indeed, the incorporation of PRP provided the collagen-GAG scaffolds with growth factors that were released from the novel constructs for up to 14 days (Figure 3). Milligrams of proteins were cumulatively released from both the composite scaffold and the PRP with similar profiles (Figure 3A), with a burst release in the first day followed by a sustained release for 14 days; however, there was more protein released from the composite scaffolds compared to PRP gel.

In terms of growth factors, VEGF (Figure 3B), bFGF (Figure 3C), PDGF-BB (Figure 3D), and TGF-β1 (Figure 3E) were successfully released from both materials for 14 days. Of note, VEGF (Figure 3B) and bFGF (Figure 3C) release presented a sustained and more intense characteristic from day 5 to day 14 in the composite scaffolds when compared to PRP gel. Finally, TGF-β1 (Figure 3E) reached a plateau within 5 days in both materials, albeit at a higher amount in the composite scaffold. These results confirmed the ability of our scaffolds to release growth factors, with positive benefits to the release profile (e.g., higher total amounts, and increased release rates) observed when incorporated within the collagen-GAG scaffold.

### Factors Released From Our Composite Scaffolds Was Able to Promote Cell Proliferation and Migration

**Figure 4:**
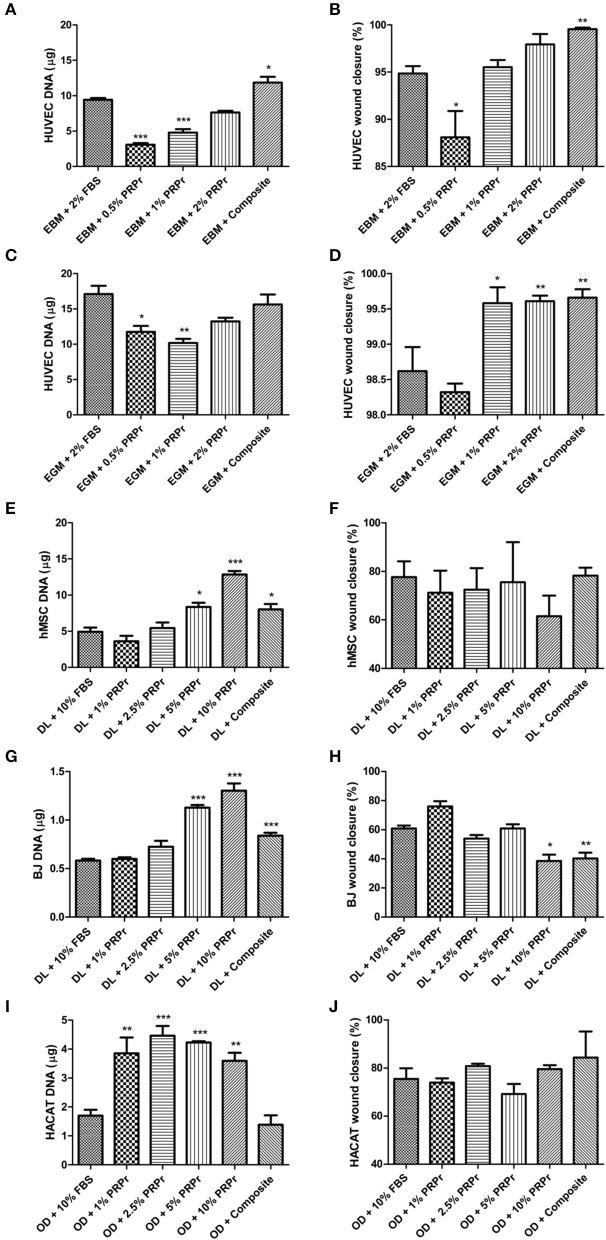
Platelet-rich plasma releasate (PRPr) and composite scaffolds sustain cell proliferation and migration. Cells were cultured in control medium supplemented with FBS or different concentrations of PRPr. Additionally, cells were cultured in FBS-free medium but with composite scaffold placed in hanging inserts. Proliferation analysis was measured by DNA quantification with picogreen assay after 4 days of culture **(A,C,E,G,I)** and cell migration was measured by percentage of wound closure in a scratch assay **(B,D,F,H,J)**. HUVEC were cultured in endothelial basal medium (EBM, which sustains cells) **(A,B)** or endothelial growth medium (EGM, which has a cocktail of factors to promote cell activity) **(C,D)**. hMSC **(E,F)**, and BJ fibroblasts **(G,H)**, were cultured in DMEM low glucose (DL). HACAT were cultured in Optimized DMEM (OD) **(I,J)**. Data are expressed as mean ± standard error of the mean. *p < 0.05, **p < 0.01, and ***p < 0.001 after one-way Anova with Dunnett's post-test analysis.

Having confirmed the ability of our composite scaffolds to release growth factors, we questioned if the factors released from our composite scaffold would be sufficient to maintain cell growth and migration capacity, even in the absence of FBS in culture medium. Additionally, we investigated PRP releasate (PRPr), which is the serum obtained after activation of the PRP gel left overnight at 4°C and centrifuged to discard the fibrin content (Do Amaral et al., 2015). PRPr was diluted in culture medium in different concentrations as a substitute to supplementation of FBS. Depending on the concentration used, PRPr could match or even outperform cell proliferation compared to control FBS. For instance, 2% PRPr matched HUVEC proliferation both in basal (Figure 4A) and growth control medium (Figure 4C); 5% and 10% PRPr induced greater hMSC (Figure 4E) and BJ (Figure 4G) proliferation; while with HACAT, all PRPr concentrations induced greater cell proliferation compared to control (Figure 4I). In a similar way, our composite scaffolds could also match cell proliferation or even increase it compared to FBS controls in all cells analyzed (Figures 4A,C,E,G,I). For HUVECs cultured in basal medium (EBM-2) our composite scaffold overcame the control proliferation rate (Figure 4A). When HUVECs were cultured in growth medium (EGM-2) the composite scaffolds could not overcome the control proliferation (Figure 4C), probably due to exogenous growth factors that already induced proliferation in the growth medium. The composite scaffolds induced greater hMSC (Figure 4E) and BJ (Figure 4G) than FBS controls, while similar proliferation in HACAT (Figure 4I).

Besides cell proliferation, we also investigated cell migration responses to our PRPr and composite scaffold. Overall, PRPr and our composite scaffold did not greatly influence cell migration (Figures 4B,D,F,H,J). For hMSC (Figure 4F) and HACAT (Figure 4J), for instance, there was no statistical difference in any of the groups. For HUVEC both in basal (Figure 4B) and growth medium (Figure 4D) although statistical significance were observed in some groups, such as in our composite scaffold, the overall difference to control was <5%. Finally, BJ migration was diminished in 10% PRPr and in our composite scaffold (Figure 4H). Cell morphology was not altered in any of the treatment groups (Supplemental Figure 1). Collectively, these data lead us to conclude that PRPr, at select concentrations, and our composite scaffold could remove the necessity for FBS supplementation in the culture of a diverse variety of cell types involved in wound healing.

### Content Released From the Composite Scaffolds Increased Endothelial Tubular Formation in Late Time-Points

**Figure 5:**
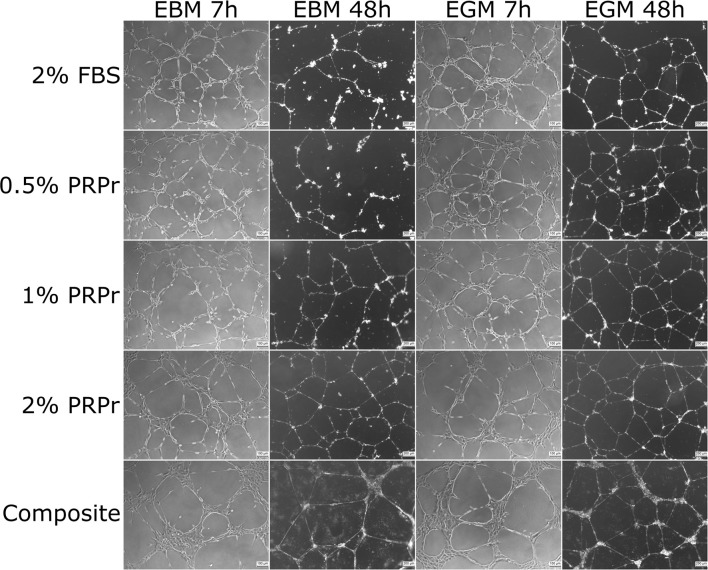
Tubule formation with platelet-rich plasma releasate (PRPr) and composite scaffold-conditioned medium. Representative images of HUVEC cultured after 7 and 48 h in growth factor-depleted Matrigel to induce tubulogenesis in the presence of basal (EBM) or growth (EGM) medium supplemented with FBS or different PRPr concentrations, as well as with a 24 h conditioned medium incubated with the composite scaffold. After 48 h in EBM it is possible to notice increased tubulogenesis in 2% PRPr and the composite scaffold compared to 2% FBS control. One hundred micrometer scale bars for 7 h pictures, and 200 μm scale bars for 48 h pictures.

**Figure 6:**
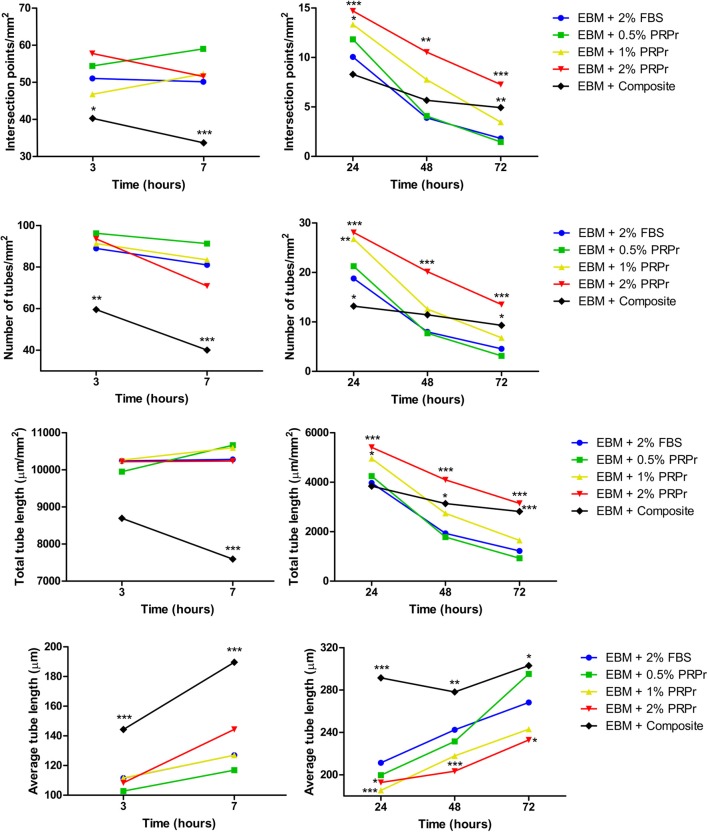
Tubule formation quantification in samples with endothelial basal medium (EBM). Intersection points, number of tubes, total tube length, and average tube length were quantified. The composite scaffold further enhanced tubule formation compared to control in later time-points. Data are expressed as mean. *p < 0.05, **p < 0.01, and ***p < 0.001 after two-way Anova with Bonferroni post-test analysis compared exclusively with EBM + 2% FBS at each time-point.

**Figure 7:**
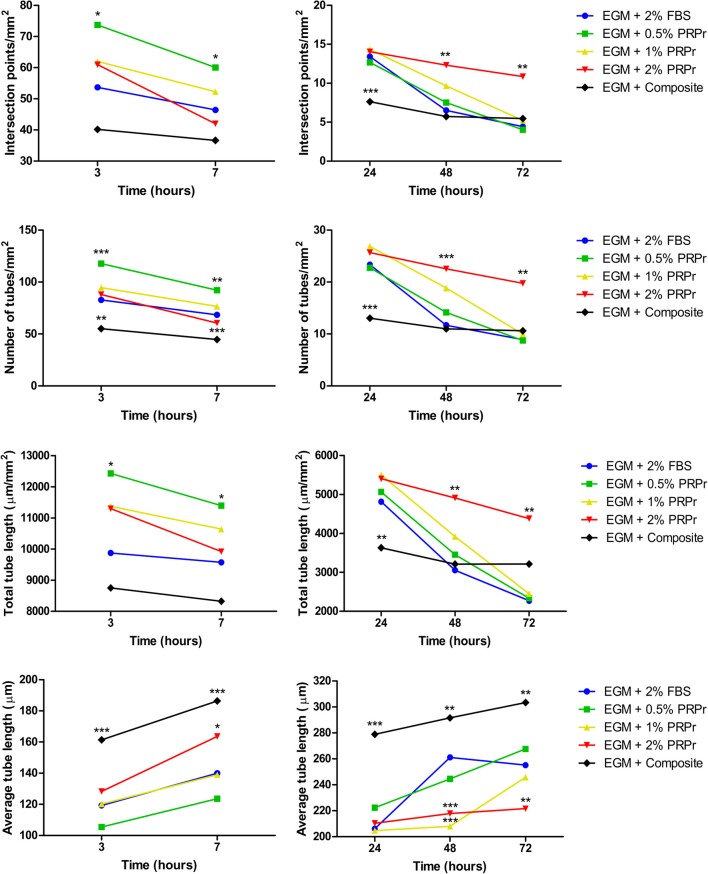
Tubule formation quantification in samples with endothelial growth medium (EGM). Intersection points, number of tubes, total tube length, and average tube length were quantified. The composite scaffold matched tubulogenesis compared to 2% FBS control. Data are expressed as mean. *p < 0.05, **p < 0.01, and ***p < 0.001 after two-way Anova with Bonferroni post-test analysis compared exclusively with EGM + 2% FBS at each time-point.

We used conditioned media from composite scaffolds incubated for 24 h in basal (EBM-2) and growth (EGM-2) medium without FBS to analyse angiogenic properties on HUVECs cultured in Matrigel. Medium supplemented with 2% FBS was used as control. In parallel, we also analyzed different concentrations of PRPr when substituted for FBS in media. Qualitative analysis of images taken after 7 and 48 h of experiment (Figure 5) already suggested that differences among groups were more noticeable in basal medium and especially at the 48-h time-point. For instance, it is possible to observe an increased tubular content with increasing concentration of PRPr and in the composite scaffolds compared to control sample with 2% FBS. Additionally, it is possible to observe a different pattern of tubule formation in the composite samples, both in basal and growth medium. At 7 h, cells were present in big clusters with fewer ramifications and apparent inferior tubule formation in composite scaffolds compared to other samples. Nevertheless, after 48 h, composite scaffolds presented well-formed tubular network. Most interestingly, the areas previously occupied by the cell clusters were junction points between the luminal-like structures lined with endothelial cells. Quantification of tubule formation (Figure 6) showed that in early time-points (3 and 7 h) composite samples presented similar or lower values of intersection points, number of tubes and total tube length compared to control, while different PRPr concentrations presented no difference compared to control when in basal medium, and 0.5% PRPr was superior to control when in growth medium (Figure 7). This pattern changed after 48 and 72 h. Composite scaffold samples presented similar or higher values of intersection points, number of tubes and total tube length compared to control, especially in basal medium. Increasing concentrations of PRPr, in particular 2% PRPr, also presented similar or higher values of intersection points, number of tubes and total tube length compared to control at all time-points. The advantage was clear after 48 and 72 h, both in basal and growth medium. Of note, the average vessel length was always superior in composite samples in both basal (Figure 6) and growth medium (Figure 7). These results suggest that our composite scaffold is inducing increased tubule formation in late time-points compared to control, and that the pattern of tubule formation in this condition is different, with the formation of cell clusters in early time-point, which develop into tubules structures with time.

### Scaffold Vascularization Was Increased in Composite Scaffolds *in vitro*

**Figure 8:**
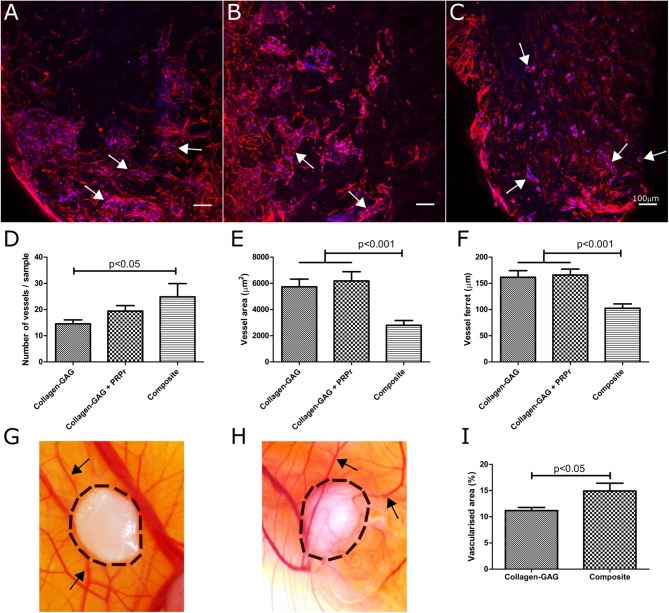
Composite scaffolds increased vascularization capacity compared to collagen-GAG scaffolds. Samples were vascularised *in vitro* **(A–F)** with a co-culture of HUVEC and hMSC for 10 days. Immunofluorescence analysis of collagen-GAG scaffolds **(A)**, collagen-GAG scaffolds cultured with platelet-rich plasma releasate (PRPr) instead of FBS **(B)** and the composite scaffold **(C)**. Actin cytoskeleton is stained in red and nuclei are stained in blue. Scale bars of 100 μm. Arrows indicate vessel-like structures. There were more vessels per samples in the composite scaffold **(D)**, although they were smaller in area **(E)** and ferret **(F)**. Collagen-GAG scaffolds **(G)** and the composite scaffold **(H)** were also vascularised *in vivo* in a chick chorioallantoic membrane (CAM) assay. Composite scaffolds were more vascularised than the collagen-GAG scaffolds **(I)**. Black arrows indicate blood vessels on the CAM and the dashed black lines indicate the area occupied by the scaffolds. Data are expressed as mean ± standard error of the mean. Statistical analysis was performed with one-way Anova with Tukey's Multiple Comparison Test in **(D–F)** and with unpaired two-tailed *t*-test in **(I)**.

To add further support to the angiogenic potential of these scaffolds, we evaluated our composite scaffold's capacity to promote scaffold vascularization. We compared the composite scaffold with the original collagen-GAG scaffolds to understand if PRP incorporation would have any effect on scaffold vascularization. Collagen-GAG scaffolds were supplemented with FBS, or with PRPr to verify if PRPr would have any effect on scaffolds vascularization. As before, the composite scaffold was kept in medium without FBS or PRPr supplementation. As expected (Mcfadden et al., 2013; Do Amaral et al., 2019), collagen-GAG scaffolds were properly vascularised after 10 days of culture (Figure 8A). By qualitatively analyzing the immunofluorescence images, the use of PRPr in collagen-GAG medium supplementation did not greatly impact scaffold vascularization compared with FBS supplementation (Figure 8B). The composite scaffolds could also be properly vascularised within the period of 10 days in culture (Figure 8C) and qualitative analysis suggested that composite scaffolds presented smaller vessel-like structures, resembling capillary-like structures, but in greater number when compared to collagen-GAG samples. Image quantification showed that composite scaffolds did indeed present statistically more vessel-like structures compared to collagen-GAG samples (Figure 8D). Interestingly, the vessel-like structures were smaller both in area (Figure 8E) and ferret (Figure 8F) in our composite scaffold compared to collagen-GAG samples. In the collagen-GAG scaffolds, there was no statistical difference between the uses of FBS or PRPr in the collagen-GAG culture, although there was a trend toward more vessel-like structures with PRPr (Figure 8D). Overall, this suggests that PRP incorporation into the collagen-GAG scaffolds increased scaffold vascularization.

### Composite Scaffolds Promoted Greater Angiogenesis *in vivo* Compared to Collagen-GAG Scaffolds

As a final way to test angiogenic/vascularization properties, we challenged our scaffolds in the CAM assay. Since the CAM is highly vascularised, and chick embryos only become immunocompetent by day 18 of development, the CAM assay has been extensively used to test the angiogenic potential of scaffolds without inducing an immune response (Azzarello et al., 2007; Fishman et al., 2013; Li et al., 2015; Woloszyk et al., 2016; Moreno-Jimenez et al., 2017; Ryan et al., 2019). In this work, we directly compared collagen-GAG (Figure 8G) with composite scaffolds (Figure 8H). In accordance with our previous *in vitro* data, the composite scaffold induced greater vascularization of its surrounding area compared to collagen-GAG scaffolds, which could be quantified (Figure 8I) as an increase of 33.53% of vascularised area. These results further supported the finding that incorporation of PRP into the collagen-GAG scaffolds enhanced their angiogenic and vascularization capacities, which are critical processes for successful wound healing.

### Composite Scaffolds Induced Anti-inflammatory Marker Secretion From Macrophages

**Figure 9:**
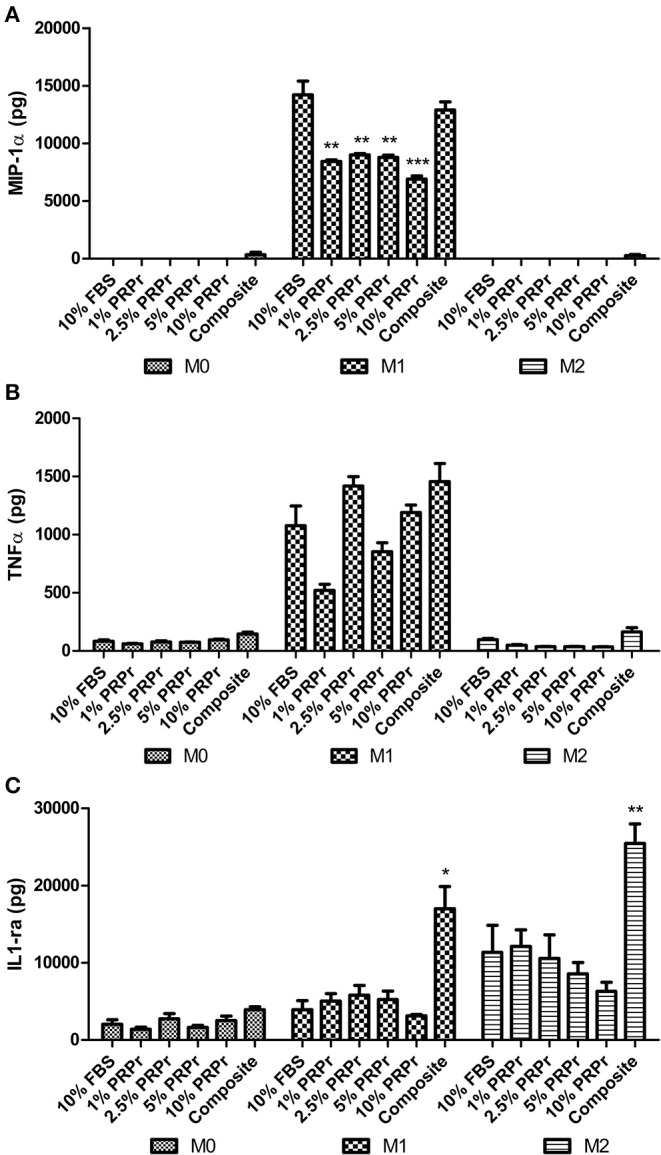
Modulation of macrophage polarization. THP1 cells were cultured for 72 h in growth medium (M0), supplemented with Interferon-γ (IFNγ), and lipopolysaccharide (LPS) (M1) or interleukin-4 and -13 (M2). Medium contained 10% FBS, varying concentrations of PRPr, or our composite scaffold placed on a cell culture insert. Culture medium was collected and MIP-1α **(A)**, TNFα **(B)**, and IL1-ra **(C)** were quantified by ELISA. High levels of MIP-1α and TNFα were observed in M1 induction **(A,B)**, although different concentrations of PRPr significantly reduced the levels of MIP-1α compared to 10% FBS control **(A)**. Treatment with the composite scaffold significantly increased the levels of IL1-ra compared to 10% FBS upon both M1 and M2 induction **(C)**. Data are expressed as mean ± standard error of the mean. *p < 0.05, **p < 0.01, and ***p < 0.001 after statistical analysis performed with one-way Anova with Dunnett's post-test.

As one of the first cell types to encounter an implanted material, macrophages play an important role in determining the acceptance of any material by the body. Macrophages polarize into different phenotypes at different stages of the wound healing cascade, varying from an initial pro-inflammatory stage, with an M1 phenotype, to a resolution stage at later time-points, with an M2 phenotype (Mantovani et al., 2013). In this study, we evaluated if the content released by our composite scaffold would differentially regulate the macrophage response in growth medium (M0) and in pro- (M1) or anti-inflammatory (M2) medium. We also evaluated if different concentrations of PRPr in cell culture media had any effect on macrophage polarization (Figure 9). Importantly, the control samples comprised of macrophages cultured in their respective medium (M0, M1 or M2) supplemented with 10% FBS. In M0 medium, no difference was observed in the secretion of pro-inflammatory factors, such as MIP1α (Figure 9A) and TNFα (Figure 9B), or the anti-inflammatory cytokine IL1-ra (Figure 9C), indicating that neither PRPr nor the composite scaffolds modified macrophage response. This indicates that neither PRPr nor our composite are affecting the baseline macrophage response. Upon M1 induction, macrophages released high levels of both MIP1α and TNFα. Interestingly, the composite scaffolds induced macrophages to release similar amounts of both MIP1α and TNFα compared to control, 10% FBS. Nonetheless, the addition of different concentrations of PRPr dampened the secretion of MIP1α (*p* < 0.01) and TNFα (not significant). These results indicate that the presence of the composite scaffolds allowed macrophages to become pro-inflammatory in the presence of pro-inflammatory factors.

Upon induction with M2 factors, macrophages released increased levels of IL1-ra in all groups, with cells treated with the composite scaffolds showing significantly higher levels (*p* < 0.01) compared to 10% FBS control. Interestingly, even upon induction with M1 factors, macrophages released significantly higher levels of IL1-ra (*p* < 0.05) compared to 10% FBS control. This suggests that factors released by the composite scaffolds have the capability to enhance the secretion of an anti-inflammatory cytokine when presented with the relevant cues. Taken together, the results suggest that PRPr and the composite scaffolds do not influence macrophage polarization *per se* (as evidenced in M0 medium). However, upon pro- or anti-inflammatory induction, the composite scaffolds are able to modulate cytokines secretion, allowing a pro-inflammatory response, which is essential for the initial stages of wound healing, and an enhanced anti-inflammatory factor secretion, which is required for the subsequent remodeling response.

### Composite Scaffolds Could Support Fibroblast and Keratinocyte Co-culture

**Figure 10:**
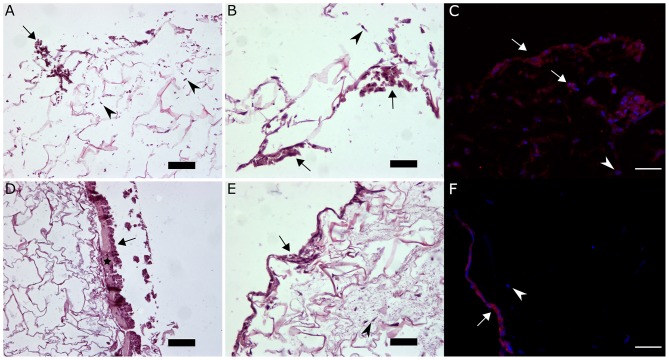
Organotypic skin culture. Collagen-GAG scaffolds **(A–C)** and the composite scaffold **(D–F)** were cultured with HACAT keratinocytes and BJ fibroblasts for 14 days. Representative images of H&E stained samples show keratinocytes (arrows) on the surface, and not inside, of the composite scaffolds adhered to fibrin layer (*) **(D,E)**. In the CG scaffolds, keratinocytes were found inside the pores of the scaffold **(A,B)**. Fibroblasts (arrowheads) were successfully cultured in both systems. Keratinocytes and fibroblasts distribution was corroborated with anti-pan-cytokeratin immunofluorescence with red-labeled cells identifying the keratinocytes (arrows) and un-labeled cells identifying the fibroblasts (arrowheads) **(C,F)**. One hundred micrometer scale bars in **(A,D)** and 50 μm scale bars in **(B,C,E,F)**.

As well as the promotion of vascularization and cell growth of the dermal layer, another key step toward a successful wound healing involves separating successful fibroblast ingrowth, proliferation and ECM production in the dermal layer from re-epithelialization by keratinocytes (Gurtner et al., 2008; Eming et al., 2014). We hypothesized that the fibrin clot may facilitate maintenance of the keratinocytes on the outer surface of the scaffold. Thus, we co-cultured fibroblasts and keratinocytes in collagen-GAG scaffolds and composite scaffolds. Fibroblasts could be successfully cultured in both materials, as expected. On the other hand, since collagen-GAG scaffolds have a porous outer surface (i.e., not a continuous, flat surface), it became clear from H&E stained samples that keratinocytes did not form a linear epidermal-like surface in the collagen-GAG scaffolds (Figures 10A,B). In some points of the scaffolds, keratinocytes would even infiltrate toward its center (Figure 10A). Alternatively, PRP incorporation into collagen-GAG scaffolds resulted in the formation of a thin fibrin layer on the surface of the composite scaffolds (Figures 10D,E), which allowed keratinocytes to attach and form a more continuous layer compared to collagen-GAG samples. Immunofluorescence against pan-cytokeratin further corroborated the H&E observations. The keratinocytes were restricted to a lining surface on the composite scaffolds (Figure 10F), whereas keratinocytes were randomly distributed in the collagen-GAG scaffold (Figure 10C).

## Discussion

The objective of this work was to improve the wound healing potential of collagen-GAG scaffolds by functionalising it with platelet-rich plasma using a simple fabrication strategy. We hypothesized that PRP would provide the scaffolds with growth factors that play important roles in wound healing, and particularly in angiogenesis and vascularization. Moreover, the fibrin polymerised after PRP activation could potentially provide a substratum for keratinocyte attachment and growth on the scaffolds' surface. These should help overcome two limitations of collagen-GAG scaffolds: (i) the long period needed for scaffolds vascularization once implanted; (ii) and the need of a two-stage surgical procedure—whereby the scaffolds are first implanted with an outer silicone layer, which is later replaced by an epidermal autograft (Shahrokhi et al., 2014). From the PRP perspective—which has shown promise for its direct use—combining it with a collagen-GAG scaffold will enhance PRP's mechanical properties, as well as potentially controlling the release of its growth factors. In this work, we successfully developed a fabrication technique to incorporate PRP homogenously within collagen-GAG scaffolds. The resulting composite scaffold matched the mechanical properties of the original collagen scaffold. From a functional perspective, growth factors were released from the resulting composite scaffold for up to 14 days; these factors ensured equivalent, or higher, proliferation of a variety of key cell types associated with wound healing (e.g., human endothelial cells, mesenchymal stromal cells, fibroblasts, and keratinocytes), when compared with FBS. Furthermore, these factors increased the composite scaffolds' angiogenic/vascularization potential, and allowed a pro-inflammatory response from macrophages while potentially enhancing an anti-inflammatory response when induced with relevant cues. Finally, the fibrin clot within the scaffold pores could support fibroblast and keratinocyte co-culture in a bilayered manner, which was not observed with the collagen scaffold.

The fabrication strategy was simplified by taking advantage of the endogenous fibrinogen polymerization potential in PRP to engineer a composite of PRP-derived fibrin within the pores of the collagen-GAG scaffolds. In practice, we envisage that PRP could be easily obtained at bedside and incorporated into the scaffolds intraoperatively using our strategy, prior to implantation. Similar approaches taking advantage of endogenous fibrinogen polymerization in PRP had been attempted with PLA scaffolds for tendon repair (Visser et al., 2010), PLGA (Lei et al., 2009), PCL (Berner et al., 2012), and chitosan (Shimojo et al., 2016) scaffolds for bone regeneration. However, to our knowledge, this work represent the first time that PRP has been incorporated into a proven and commercially available porous regenerative template, the collagen-GAG scaffolds, for skin tissue engineering. Previous attempts of platelet derived products have been tested in skin tissue engineering [e.g., chitosan and hyaluronic acid dressings were mixed with platelet lysate (Rossi et al., 2013); collagen/gelatin scaffold was impregnated with platelet lysate solution (Ito et al., 2013), and collagen type I gel was mixed with PRP (Houdek et al., 2016)] with promising outcomes, adding further support to this approach.

Although PRP has many promising benefits for wound healing, PRP in a gel form on its own suffers from poor mechanical properties, which is believed to limit its translational success (Sell et al., 2012). Alternatively, our composite scaffold acquired similar mechanical properties to the original collagen-GAG scaffold, which are superior to PRP gel. Similar findings were observed with increased mechanical properties of electrospun PRP incorporated into PCL, PGA and silk fibroin compared to PRP only fibers (Sell et al., 2011). In a previous work from our group, fibrin gel mechanical properties were also improved when incorporated into collagen-GAG scaffolds (Brougham et al., 2015). Furthermore, our tribology data showed superior adhesiveness characteristics of our composite scaffold compared to wet collagen-GAG scaffolds. In skin applications, where the tissue-engineered construct is submitted to different movements and mechanical pressure, enhancing mechanical properties and adhesiveness are important features (Salamone et al., 2016; Qu et al., 2018).

Having analyzed the structural and mechanical properties, we showed that PRP incorporation provided the collagen-GAG scaffolds with a cocktail of factors that could efficiently improve their wound healing potential. By soak-loading the collagen-GAG scaffolds in calcium chloride, which triggers endogenous thrombin formation, we were able to activate platelets, which resulted in growth factors being released for up to 14 days. Indeed, it has been shown that platelets activated with calcium release growth factors over 7 days, while with exogenous thrombin almost 100% of the growth factors are released after 1 h (Marx, 2001; Foster et al., 2009). Accordingly, we propose that as a simple, cost-effective and straightforward method prior to implantation, collagen-GAG scaffolds are soak-loaded in calcium chloride and then in patient-derived PRP to provide a composite scaffold for clinical use. Although it is well-known that platelets can be activated when in contact with collagen (Fufa et al., 2008; Harrison et al., 2011), the current version of the collagen-GAG scaffold was not enough to promote platelet activation and fibrin polymerization.

The composite scaffolds released VEGF, bFGF, and PDGF-BB—key factors in wound repair—for up to 14 days, at higher levels and higher rates than those observed for PRP gels. This could be explained by a possible increased degradation of the fibrin network at these time-points in the composite scaffold, which was not observed in the more compact fibrin network of the PRP gel; or by initial interactions of the growth factors with the collagen-GAG matrix followed by a delayed release after 5 days. Indeed, chondroitin sulfate may bind to different growth factors and cytokines (Mizumoto et al., 2013; Djerbal et al., 2017). Moreover, the PRP gel has a compact shape with thick fibrin fibers as described in the histological analysis. On the other hand, the composite scaffold has a discoid shape with thin fibrin fibers within pores. This looser distribution of the fibrin may provide more surface area to allow a higher release of entrapped PRP factors. Other approaches that incorporated PRP into hydrogels to better control the release of growth factors were also successful in improving the regenerative potential of PRP in different applications (Lu et al., 2008, 2019; Bir et al., 2011; Kurita et al., 2011; Yang et al., 2011; Qiu et al., 2016; Jain et al., 2017; Liu et al., 2017).

The effects of the released factors were evaluated for both our composite scaffold and PRP releasate (PRPr) in different cell types (endothelial, mesenchymal stromal cells, macrophages, fibroblasts, and keratinocytes) since all of them have key roles during different stages of wound healing (Gurtner et al., 2008; Eming et al., 2014). There is vast literature suggesting that PRP supports endothelial cell proliferation and induces angiogenesis/vascularization both *in vitro* and *in vivo* (Kakudo et al., 2014; Anitua et al., 2015; Notodihardjo et al., 2015; Etulain et al., 2018; Romaldini et al., 2019; Samberg et al., 2019). Our work corroborates those findings, but, importantly, shows that this activity is maintained within a biomaterial scaffold with proven regenerative potential. Particularly, our work shows that our composite scaffold was able to promote angiogenesis and vascularization both *in vitro* and *in vivo* in the absence of FBS supplementation. Before incorporating PRP within the scaffold, we observed that PRPr at the concentration of 2% outperformed 2% FBS when combined with basal medium in tubule formation assays, demonstrating that PRP provides growth factors necessary to promote angiogenesis. PRPr at the concentration of 2.5% promoted hMSC proliferation similarly to 10% FBS, as previously shown by our group (Do Amaral et al., 2015), while our composite scaffold outperformed 10% FBS. Similar results were observed with fibroblasts, also corroborating literature findings demonstrating PRP's capacity to promote fibroblast proliferation (Liu et al., 2002; Graziani et al., 2006; Anitua et al., 2009; Cho et al., 2012; Kushida et al., 2013; Wang et al., 2019). On the other hand, 10% PRPr and our composite scaffold decreased the migration of fibroblasts, consistent with others' observations (Xian et al., 2015). Regarding keratinocytes, even in very low concentrations of PRPr (i.e., 1%), were able to induce cell proliferation to a greater extent compared to 10% FBS control and our composite scaffold matched 10% FBS proliferation. In previous work, PRP only at the concentration of 20% was able to overcome keratinocyte proliferation compared to 10% FBS; and similar to our work, PRP did not affect keratinocyte migration (Xian et al., 2015).

In regards to inflammation, the content released by our composite scaffold as well as PRPr treatment did not interfere with macrophage polarization *per se*, i.e., without an induction toward M1 or M2 phenotype. During physiological wound healing, macrophages drift from the initial pro-inflammatory M1 phenotype toward a resolution M2 phenotype over time (Mantovani et al., 2013). Therefore, a robust response from macrophages is essential in order to ensure that their function is fulfilled in all stages of the wound healing process. On the other hand, in chronic wounds, where inflammation persists, macrophages are found mainly in M1 pro-inflammatory phenotype (Krzyszczyk et al., 2018). Also, it has been established that PRP contains both pro and anti-inflammatory cytokines (Amable et al., 2013). In our study, we showed that the presence of PRPr and the composite scaffold did not modify the macrophage response in growth (M0) medium. However, when macrophages were challenged with pro- or anti-inflammatory factors (M1 and M2 medium, respectively), the secretion of both pro- (MIP1α and TNFα) and anti-inflammatory (IL1-ra) cytokines were modulated; PRPr dampened the production of MIP1α and the content released from our composite scaffolds allowed macrophages to secrete both MIP1α and TNFα to the same levels as 10% FBS, while increasing the level of IL1-ra secretion. Indeed, anti-inflammatory effects of PRP have previously been reported in different applications (Zhang et al., 2013; Moussa et al., 2017; Abdul Ameer et al., 2018); although other groups reported stimulation of both M1 and M2 markers (Escobar et al., 2018). The study of macrophage polarization is an evolving field, with several cytokines and markers expressed by subpopulations that may go beyond the classical M1/M2 and pro-/anti-inflammatory phenotypes (Chávez-Galán et al., 2015). Therefore, further work is needed to fully elucidate the role of PRP and our composite scaffold in inflammation, particularly in the context of wound healing.

Finally, we were able to show that our composite scaffold was able to support fibroblast and keratinocyte co-culture *in vitro* and to isolate each cell type to a bilayer. The thin fibrin layer on the surface of the composite allowed keratinocytes to grow forming a surface layer, which was not observed in the original collagen-GAG scaffolds. Although keratinocytes do not adhere directly to fibrin or fibrinogen due to lack of alpha v beta 3 integrin (Kubo et al., 2001), fibrin can promote keratinocytes migration (Drukala et al., 2001) by exposing plasminogen (Geer and Andreadis, 2003) and keratinocytes are able to digest and invade a fibrin matrix (Ronfard and Barrandon, 2001). We believe that restricting the keratinocytes to a surface layer and raising them to an air-liquid interface helps drive the bilayer formation.

Future work will aim to address some of the limitations in this study. Our *in vivo* analysis, performed with the CAM assay, focused on angiogenesis/vascularization. Future preclinical data on animal wound healing models, both in acute and chronic scenario, are needed to corroborate our current findings. Additionally, as a more complete picture of PRP emerges, a deeper investigation into the molecular aspects of the interactions should help enhance our knowledge and use of PRP in wound healing.

## Conclusions

We successfully incorporated PRP into collagen-GAG scaffolds in a simple method that could be easily translated to the clinical scenario where autologous PRP could be obtained from patient blood and incorporated into the collagen scaffold prior to implantation into a wound. The composite scaffold presented enhanced mechanical properties compared to PRP gel, while the PRP provided the scaffold with growth factors that were released for up to 14 days. The released content was able to match, or overcome, cell proliferation in a variety of key cells involved in wound healing, when compared to standard FBS culture *in vitro*. Finally, our composite scaffold presented increased angiogenic and vascularization capacity *in vitro* and *in vivo* as well as the capacity to support a fibroblast and keratinocyte co-culture *in vitro*, and allowed a pro-inflammatory response while enhancing an anti-inflammatory cytokine secretion from macrophages when induced by select cues. We expect that future *in vivo* preclinical data will further support the use of our composite scaffold to promote wound healing more efficiently than the original collagen-GAG scaffolds.

## Data Availability Statement

The raw data supporting the conclusions of this manuscript will be made available by the authors, without undue reservation, to any qualified researcher.

## Ethics Statement

The studies involving human participants were reviewed and approved by Royal College of Surgeons in Ireland Research Ethics Committee (REC1463b). The patients/participants provided their written informed consent to participate in this study. Ethical review and approval was not required for the animal study because Chick Chorioallantoic Membrane (CAM) assay was used to investigate scaffold's angiogenic potential *in vivo*. All experimentation carried out on chick embryos was in accordance with the EU Directive 2010/63/EU for animal experiments. The CAM assay does not require ethical review and approval.

## Author Contributions

RA contributed to the conception and development of the idea, designing, performing and analyzing experiments, and writing the manuscript. NZ contributed to cell culture and samples preparation. EP contributed to the mechanical properties experiments. BC contributed to microscopical imaging. CH contributed to the scanning electron microscopy. FS, CS, and CM contributed to the tribology experiments. RS contributed to the macrophage experiments. AG-V contributed to the CAM assay. BO'S contributed to the conception and development of the idea with his clinical expertise. FO'B and CK contributed to the conception and development of the idea, designing and analyzing the experiments, and writing the manuscript. All authors provided critical feedback and helped shape the research, analysis, and manuscript.

### Conflict of Interest

The authors declare that the research was conducted in the absence of any commercial or financial relationships that could be construed as a potential conflict of interest.

## Supplementary Material

Supplemental Figure 1 Monolayer cell culture. Representative images of monolayers after 4 days in culture. HUVEC were cultured in endothelial basal medium **(D,I,N,S,X)** supplemented with 2% FBS **(D,E)**, 0.5% PRPr **(I,J)**, 1% PRPr **(N,O)**, 2% PRPr **(S,T)**, or composite scaffold in an insert **(X,Y)**. hMSC **(A,F,K,P,U,Z)** were cultured in DMEM low glucose, HACAT **(B,G,L,Q,V,AA)** were cultured in Optimized DMEM and BJ **(C,H,M,R,W,AB)** were cultured in DMEM low glucose supplemented with 10% FBS **(A–C)**, 1% PRPr **(F–H)**, 2.5% PRPr **(K–M)**, 5% PRPr **(P–R)**, 10% PRPr **(U–W)**, or composite scaffold in an in insert **(Z,AA,AB)**. Cell morphology was maintained similar across the different treatments. One hundred micrometer scale bars.
