# Supplementary figures and images for: Functionalising Collagen-Based Scaffolds With Platelet-Rich Plasma for Enhanced Skin Wound Healing Potential
Source: Front Bioeng Biotechnol. 2019 Dec 3;7:371. doi: 10.3389/fbioe.2019.00371 (PMC6915093; doi:10.3389/fbioe.2019.00371)

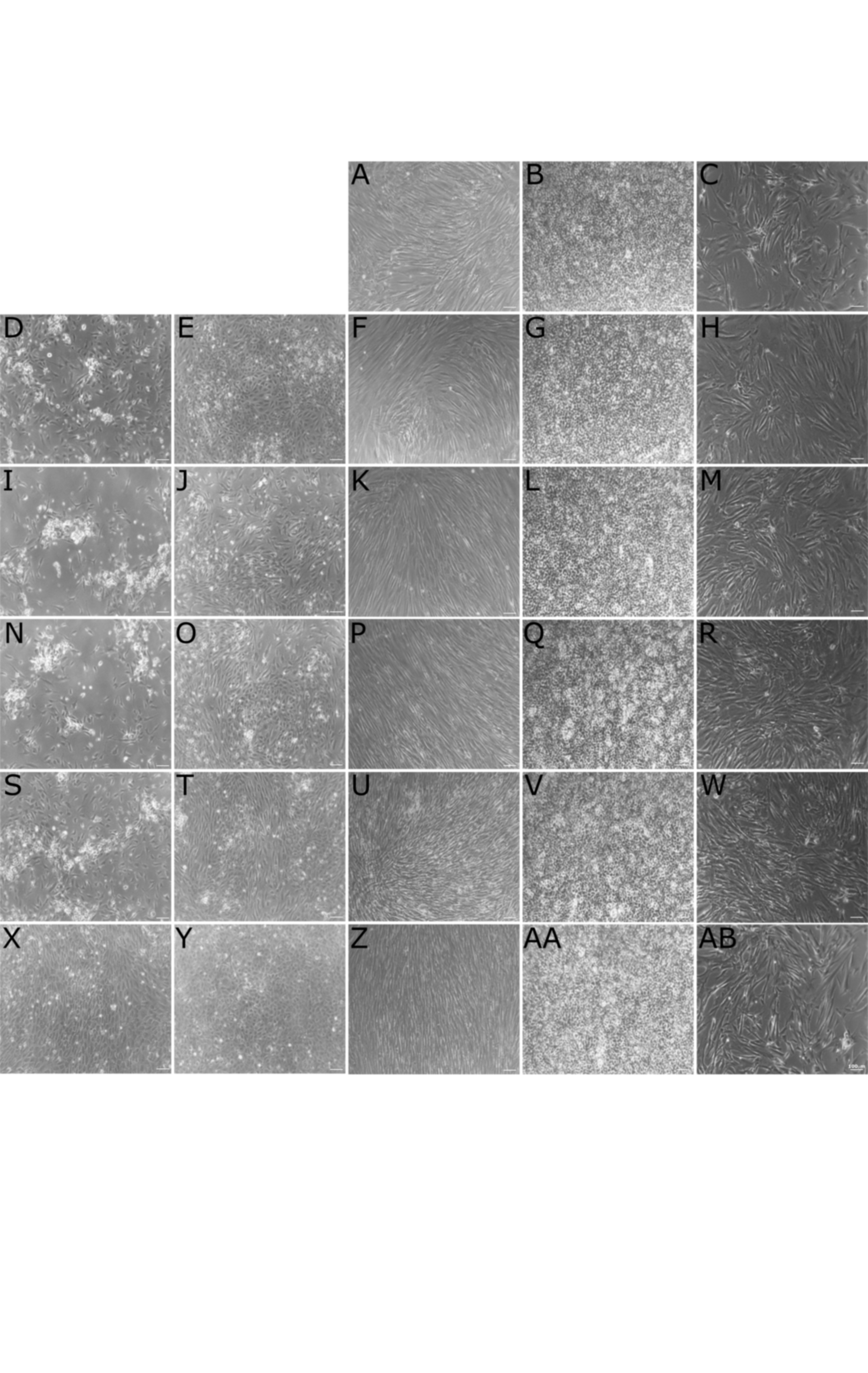

Supplement: Supplemental Figure 1 — Monolayer cell culture. Representative images of monolayers after 4 days in culture. HUVEC were cultured in endothelial basal medium (D,I,N,S,X) supplemented with 2% FBS (D,E), 0.5% PRPr (I,J), 1% PRPr (N,O), 2% PRPr (S,T), or composite scaffold in an insert (X,Y). hMSC (A,F,K,P,U,Z) were cultured in DMEM low glucose, HACAT (B,G,L,Q,V,AA) were cultured in Optimized DMEM and BJ (C,H,M,R,W,AB) were cultured in DMEM low glucose supplemented with 10% FBS (A–C), 1% PRPr (F–H), 2.5% PRPr (K–M), 5% PRPr (P–R), 10% PRPr (U–W), or composite scaffold in an in insert (Z,AA,AB). Cell morphology was maintained similar across the different treatments. One hundred micrometer scale bars. [file Image_1.TIF]
